# Molecular Regulatory Mechanisms in Chicken Feather Follicle Morphogenesis

**DOI:** 10.3390/genes14081646

**Published:** 2023-08-18

**Authors:** Gaige Ji, Ming Zhang, Yunjie Tu, Yifan Liu, Yanju Shan, Xiaojun Ju, Jianmin Zou, Jingting Shu, Zhongwei Sheng, Hua Li

**Affiliations:** 1 Key Laboratory for Poultry Genetics and Breeding of Jiangsu Province, Chinese Academy of Agricultural Science, Institute of Poultry Science, Yangzhou 225125, China; 2 School of Life Science and Engineering, Foshan University, Foshan 528231, China

**Keywords:** feather follicle, molecular mechanisms, morphogenesis, chicken

## Abstract

In China, the sale of freshly slaughtered chickens is becoming increasingly popular in comparison with that of live chickens, and due to this emerging trend, the skin and feather follicle traits of yellow-feathered broilers have attracted a great deal of research attention. The feather follicle originates from the interaction between the epidermis and dermis in the early embryonic stage. Feather follicle morphogenesis is regulated by the Wnt, ectodysplasin (Eda), epidermal growth factor (EGF), fibroblast growth factor (FGF), bone morphogenetic protein (BMP), sonic hedgehog (Shh), Notch, and other signaling pathways that exist in epithelial and mesenchymal cells. The Wnt pathway is essential for feather follicle and feather morphogenesis. Eda interacts with Wnt to induce FGF expression, which attracts mesenchymal cell movement and aggregates to form feather follicle primordia. BMP acts as an inhibitor of the above signaling pathways to limit the size of the feather tract and distance between neighboring feather primordia in a dose-dependent manner. The Notch/Delta pathway can interact with the FGF pathway to promote feather bud formation. While not a part of the early morphogenesis of feather follicles, Shh and BMP signaling are involved in late feather branching. This review summarizes the roles of miRNAs/lncRNA in the regulation of feather follicle and feather growth and development and suggests topics that need to be solved in a future study. This review focuses on the regulatory mechanisms involved in feather follicle morphogenesis and analyzes the impact of SNP sites on feather follicle traits in poultry. This work may help us to understand the molecular regulatory networks influencing feather follicle growth and provide basic data for poultry carcass quality.

## 1. Introduction

The yellow-feathered broiler, also known as the quality broiler, encompasses local chicken breeds in China or new crossbreeds from local chicken varieties. China ranks second in chicken meat production in the world [1]. In comparison with the white-feathered broiler, the yellow-feathered broiler is more popular with consumers in East Asia due to its higher meat quality and particular flavor [2,3]. Yellow-feathered broilers are usually sold as live chickens, and features such as feather color, shape, and integrity or comb shape have become criteria for consumers to judge quality. However, the market for yellow-feathered broilers is gradually moving away from the sale of live birds and toward the sale of slaughtered birds due to the influence of COVID-19 and avian influenza prevention. Thus, traits such as plumage color and physical appearance, which originally represented the quality of the breed, are no longer relevant to the consumer. The density and the size of the feather follicles at the market stage, which can affect the carcass appearance of broilers, have received increased attention from both consumers and breeders [4]. In addition, the density of feather follicles is linked closely to feather quantity, which is a valuable factor in the sale of ducks and geese [5,6].

**Figure 1:**
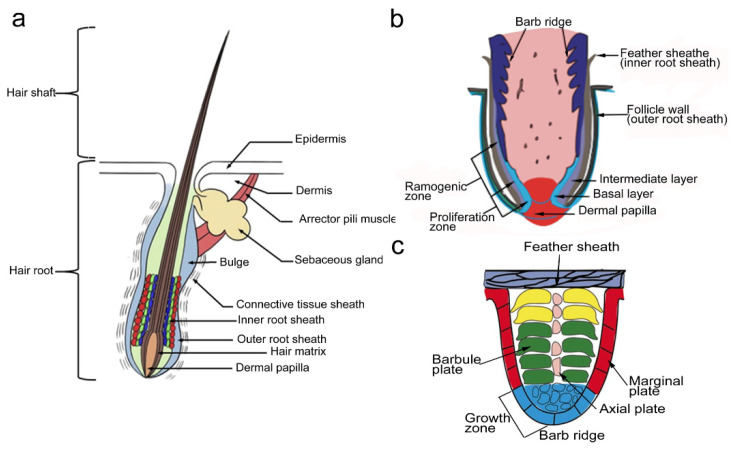
Diagram of the hair follicle (from Lin et al., 2022 [8]) (**a**), feather follicle structure (from Yu et al., 2002 [9]) (**b**), and feather barb ridge (from Yu et al., 2002 [9]) (**c**).

Feathers are derived from feather follicles, which can produce feathers of diverse colors and shapes with different functions during different physiological periods. The basic structure of a feather follicle is similar to that of a hair follicle in mammals (Figure 1). However, the structure and development of feather follicles are more complex than hair follicles due to the formation of branch ridges, feather axis ridges, and feather barbules on the interior [7] (Figure 1). The embryonic stage is an important period for feather follicle morphogenesis and also determines the number and position of feather follicles. Afterward, the type, size, or color of feathers grown from the feather follicles may change, but the number of follicles within the feather tracts is determined. In this review, we primarily focus on feather follicle formation in chicken skin. Understanding and influencing feather follicle formation are of great significance to the broiler industry. Therefore, investigating the molecular regulatory mechanisms of morphogenesis in feather follicles and the application of molecular marker-assisted selection is important for improving the carcass appearance of slaughtered and chilled yellow-feathered broilers.

## 2. Feather Follicle Development in Chickens

### 2.1. Feather Follicle Morphogenesis at the Embryonic Stage

The initiation of feather follicle primordium results from the condensation of dermal mesenchymal cells below the epidermal placode. First, the dermal precursor cells migrate to the subectoderm region and form different skin regions (macro-patterning). Then, feather follicle primordia are induced to form epidermal placodes and dermal condensations within the skin regions. The feather follicle primordium positioning process in particular areas during embryonic development is also called micro-patterning. The arrangement of feather tracts begins on embryonic day 6 (E6), resulting in bare spaces between the tracts of the chicken embryo. These bare spaces remain unfeathered throughout a chicken’s life cycle; however, in geese, these bare spaces are filled with small fluffy feathers. We can observe the obvious feather follicle primordium emerging in the dorsal midline of the chicken embryo on embryonic day 8 (E8). The dorsal skin displays clear protrusions across the feather tract, which emerge on embryonic day 9 (E9) (short buds).

**Figure 2:**
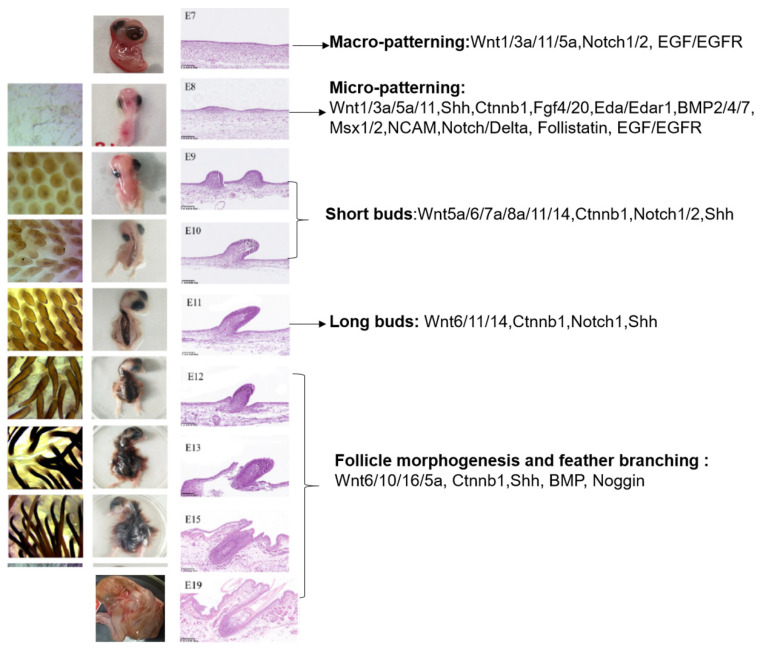
Histological sections and signaling molecules involved in the regulation of feather follicle morphogenesis in chick embryos. (Modified from Xie et al. [10].)

Histological measurements have demonstrated that feather follicle development mainly occurs between embryonic days 7 and 15 (E7–E15) in chicken embryos [10] (Figure 2). The skin shows protrusions on embryonic days 7–9 (E7–E9), and the feather buds appear along the body in an anterior–posterior direction on E10 (short buds) and E11 (long buds). At E12, the epithelium is invaginated into the underlying dermis to form the follicle structure and demarcates the boundary of a feather follicle. The epidermis is further invaginated, accompanied by the growth of dermal papilla from the dermal pulp, until feather follicle morphogenesis is completed on E15. There may be differences in time regarding feather follicle initiation and completion between different breeds, but the overall developmental process is similar. A mature feather follicle includes a mesenchymal compartment (dermal pulp cell and their underlying dermal papilla) and epithelial structures (feather and follicle sheaths), which are connected by muscles, nerves, and blood. The epithelial stem cells are stored at the collar bulge. Cells in the proliferative zone form radial and barb ridges, which eventually develop into feather barbs and barbules (Figure 1b,c and Figure 2).

### 2.2. Molecular Signaling in Feather Follicle Development

#### 2.2.1. Wnt Signaling Pathway

The interaction between epidermal and dermal mesenchymal cells is essential for feather follicular morphogenesis. Isolation and recombination experiments have demonstrated that dermal mesenchymal cells determine the direction of skin appendage development [11]. The specific orientations and sizes of the feather follicles are determined and regulated by the cell migration and a series of key molecule interactions that are present in the skin tissues. The Wnt pathway is the first signaling pathway to initiate feather follicle development by thickening epithelial cells in order to form the placode. Wnt pathways secrete glycoprotein, with more than 19 members of the family having been identified so far. According to differences in ligands and downstream events, Wnt signaling pathways are broadly divided into canonical and non-canonical Wnt pathways. Canonical Wnt signaling pathways mainly include Wnt proteins, Frizzled proteins (FZDs), β-catenin (Ctnnb1), the nuclear Lef/Tcf transcription factor family protein, and the disheveled (DSH) receptor family protein. Wnt members bind to the FZDs, which cooperate with the low-density lipoprotein receptor LRP5/6 and transmit signals to DSH. DSH inactivates the complex composed of glycogen synthase kinase-3 (GSK-3)/APC/Axin, then stabilizes Ctnnb1 to avoid ubiquitin-dependent degradation in the cytoplasm. The stabilized Ctnnb1 is transferred to the nucleus and binds to intranuclear lymphoid-enhancing factor (Lef)/transcription factor 4 (Tcf4) transcription factors. This situation activates downstream target genes involved in cell proliferation and migration [12]. 

The Wnt signal expressions change in dynamic patterns and induce the formation of feathers at various stages. The Wnt1 class (encompassing Wnt1, Wnt2, Wnt3, Wnt3a, Wnt8a, and Wnt14) functions through the canonical Wnt/β-catenin signaling pathway. Wnt1 and 3a, which activate the classical Wnt signaling pathway expressed throughout the feather tract (E6), are restricted to the placode epithelium (E8) and are related to the size of the spinal tract size [13]. Wnt8a and 14 are expressed throughout the short bud epithelium. In the long bud stage, the expression of Wnt14 is restricted to the proliferation zone. The high expression of the Wnt pathways can improve the accumulation of Ctnnb1, which is the central mediator of the Wnt/β-catenin signaling pathway. Ctnnb1 initiates the dense dermis formation, placode development, and intra-bud patterning, as well as the regulation of the polarized outgrowth of the bud (Figure 2). At the beginning of feather follicle formation, Ctnnb1 is the hub gene-regulated gene network that controls skin appendage types [14]. The overexpression of Ctnnb1 can induce feather bud formation from the foot scale skin region of chicken embryos [15]. The in ovo injection of a Wnt activator significantly increases Ctnnb1/Lef1 gene expression levels and feather follicle development in ducks and geese [16,17]. Unlike SFRP1, which regulates Wnt/β-catenin activity during hair follicle development [18,19], DKK1 as a Wnt inhibitor exhibited strong signals during the development of feather primordia and buds in geese embryos [20]. The in ovo injection of the Wnt inhibitor Dickkopf 1 (DKK1) in chicken eggs significantly decreased the feather follicle density and diameter of chicken embryos, accompanied by significantly decreased expression of the Ctnnb1/TCF4 gene [21]. A study of mice found that the Wnt and DKK pathways are key determinants in the regulation of hair follicle density with a reaction–diffusion mechanism [22]. Microarray results have also shown that the Wnt and DKK1 pathways, among others, are involved in the regulation of feather follicle density in a variety of chicken skin sections [23]. Whether Wnts and DKK1 participate in the adjustment of density differences between different species needs to be further verified in terms of feather follicles.

Non-canonical Wnt signaling is initiated by the Wnt5a type (encompassing Wnt4, Wnt5a, Wnt5b, Wnt6, Wnt7a, and Wnt11), defined as Wnt/planar cell polarity (PCP) or Wnt/Ca^2+^ pathways, which act independently of the Ctnnb1 pathway [24]. Wnt5a and 11, which trigger the JNK pathway, are initially expressed in the epithelium and mesenchyme, respectively, then disappear in the developing bud primordia and are restricted to the interbud area during placode formation. Wnt6 and 5a are expressed in the posterior compartment of the developing bud during the early short bud stage [25]. The overexpression of Wnt 6 induced outgrowths from the base, axle, or tip of the developing feathers [25]. Wnt11 is expressed in the distal feather mesenchyme and the interbud epithelium. Wnt7a is expressed in the posterior–proximal placode epithelium that is involved in the determination of feather bud orientation [26]. The sequencing results suggest that the non-canonical Wnt signaling pathway is involved in limiting the direction of feather growth via differential expression in chicken embryo integument morphogenesis [27]. The high expression of calcium signaling can reduce Ctnnb1 activity, leading to a reduction in Ctnnb1-associated feather bud formation in early skin development. PCP signaling is critical for the proximodistal orientation of wing feathers and their alignment in avian skin [28]. However, there is little research on the molecular regulatory mechanisms of non-Wnt signals for regulating feather follicle development. Further, more in-depth research is needed in order to study the relationships between non-canonical Wnt signaling and other signaling pathways.

#### 2.2.2. EGF Signaling Pathway

The epidermal growth factor (EGFs) family acts by binding to its tyrosine kinase receptor (EGFR), causing the phosphorylation of tyrosine residues and activating downstream-signaling pathways, such as the Jak, SRC kinase, and ERK pathways, etc. [29]. EGF signaling plays an important role in regulating cell proliferation, differentiation, invasion, and migration in epithelial cells. EGF/EGFR is expressed in the epidermis and dermis before the formation of the follicle placodes and maintained in the interbud regions during and after placode formation [30]. The EGF signaling is independent of the BMP pathway in the regulation of interbud development. Studies concerned with the EGF signal regulation of subsequent feather follicle development are rare. The relationships of EGF signals with other signaling pathways remain unknown.

#### 2.2.3. FGF Signaling Pathway

The fibroblast growth factor (FGF) family is composed of eighteen secreted proteins (FGFRs) and four tyrosine kinase FGF receptors (FGFRs). The activated FGFRs phosphorylate specific tyrosine residues that are coupled with downstream signals, which include the RAS-MAPK, PI3K-AKT, PLCγ, and STAT pathways [31]. FGFs are involved in cell proliferation and morphogenesis in the earliest stages of embryonic development. FGFs can initiate feather placode formation and enhance feather density in developing chicken skin [32]. A mutation of FGF20, which encodes highly conserved motifs among various species, leads to a loss in feathers, foot scales, and spur phenotypes in chickens because the mutations results in the condensation of the mesenchymal cell, which means that the epidermal placode cannot be properly formed. FGF signaling acts as an activator that drives the pattern formation of feather primordia during avian embryonic development. 

#### 2.2.4. EDA/EDAR Signals

The ectodysplasin pathway, including ectodysplasin, a secreted signaling molecule of the TNF superfamily, its receptor (EDAR), and the downstream death domain adaptor (EDARADD), plays an important role in the formation of epidermal appendages in mammals [33,34]. The EDA/EDAR signals can be activated by β-catenin and then induce FGF20 expression, triggering the formation of the feather array and tract [35,36]. The lost function of the EDA/EDAR signals causes the skin to be covered with feather primordia in its entirety, like the process of hair follicle formation in mammalian embryos. The NF-κB pathway has been identified as the main downstream signals of the EDA/EDAR pathway. The modulation of EDAR and NF-κB activity alters feather bud shape and growth or feather number and size in a developmentally specific stage [37]. The interference of EDA or EDAR can cause significant changes in the expression of many genes, including the BMP, Wnt/β-catenin, TGF-β, and Notch signal pathways, as demonstrated in studies on hair follicles [38]. Studies have revealed that the sequence and function of Edar are conserved across the vertebrate classes [35,39]. The molecular mechanism of the EDA/EDAR pathway in hair follicles may provide a reference for future works on chickens.

#### 2.2.5. BMP Signaling Pathway

Bone morphogenetic proteins (BMPs) are the members of the secreted signaling molecules transforming growth factor (TGF)-β superfamily and function by binding to specific BMP receptors (BMPRIA and BMPRIB). The canonical Smad and non-canonical Smad signaling pathways (PI3K/Akt, MAPKs, JNK, p38, and Erk) are the down-signal transduction pathways of BMPs [40,41]. BMPs play multiple roles, including cell proliferation, differentiation, apoptosis, and migration, in vertebrate tissue development. In most studies, BMPs are considered to play inhibitory roles in the feather follicle morphogenesis process. The increased expression of BMPRIA or BMPRIB inhibited feather formation and caused the expanded expression of Msx1, Msx2, and Fgfr2 throughout the maxillary mesenchyme [42]. However, some results are contradictory. The activation or repression effect may depend on the concertation of the BMPs. A high concentration of BMP4 leads to the inhibition of feather formation [43], but a low concentration of BMP4 induces feather formation through the activated downstream targeting of the Msx1 and cDermo1 genes and leads to Ctnnb1 expression [44]. Studies have also found that BMP2 and BMP7, expressed in both the epidermis and dermis, exert opposing actions on cell condensation and feather patterning through the reaction–diffusion system [45]. 

BMPs also repress various signaling pathways, including Wnt, Eda, and FGF, in order to control the formation of feather follicles. In the initial stages of feather development, the expression wave of Ctnnb1/EDA induces FGF20 expression, thereby triggering the mesenchymal cell condensation and feather patterning formation. The treatment of BMPs reduces the amount of feather primordium in a dose-dependent manner through the suppression of FGF20 expression but cannot eliminate cell aggregation [36]. FGF4 and Shh are strongly expressed in the epithelium and induce the local expression of BMP2 and 4 in periodic feather primordia formation, while BMP2 and 4 suppress their local expression [43]. The local application of the BMP antagonist Follistatin limits the inhibitory effect of BMPs, allowing the placode to develop feather buds in the feather tract regions [46]. BMPs are also involved in the formation of feather rachis and barb branching by balancing the antagonistic effect between BMPs and the BMP antagonist Noggin [9].

#### 2.2.6. Shh Signaling Pathway

The sonic hedgehog (Shh) is a secreted protein that belongs to the highly conserved hedgehog (Hh) family. In birds, Shh signals play crucial roles in feather follicle formation from initiating dermal condensation to feather filament shaping. Shh binds its receptor Patched (Ptc), leading to the activation of the seven-pass smoothened transmembrane protein (Smo) [47]. Then, the downstream transcription factor Gli1/2/3 activates the transcription of its target gene. During the early stages of feather bud development, Shh can be detected in the epidermis after the formation of the dermal condensation. The inhibition of Shh signaling leads to decreased dermal condensation, accompanied by an increase in the spatial expansion and gene expression levels of the BMP and Wnt genes [48]. Functional disruption experiments reveal that Shh cooperates with Wnt signaling to upregulate Connexin-43 expression, trigger Ca^2+^ channels, and coordinate mesenchymal cell movements to enhance feather bud elongation [49]. Fgf4, Shh, and Ptc are expressed earlier than BMP2/4 in the primordia and then disappear in the interbud regions [43]. Shh is expressed in the tip and distal end of the feather bud epithelia in the short and long bud stages, respectively [43]. The effect of ectopic Shh expression indicates that Shh is involved in the determination of the feather bud posterior–distal direction [26]. During the stage of the formation of the feather buds, Shh is expressed in the basal layer of the placode epithelium between barb ridges. BMP signaling negatively regulates Shh expression and functions as an inhibitor of feather barb ridge formation and differentiation and feather branching [50,51]. 

#### 2.2.7. Notch Signaling Pathway

Notch signaling is evolutionarily conserved and affects the development process of differentiation, proliferation, and apoptosis in many different tissues of vertebrates and invertebrates [52,53,54]. The core Notch pathway contains a set of signal transmitting chains: Notch ligands, Notch receptors, and co-ligands. A canonical Notch ligand interacts with a Notch transmembrane receptor in an extracellular space to release the Notch intracellular domain through the proteolytic cleavage of the receptor. The Notch intracellular domain then translocates to the nucleus where it binds to CBF1/Suppressor of Hairless/LAG-1 (CSL) family DNA-binding protein to activate the transcription of the target gene [53,55]. Early results suggest that the asymmetrical expressions of Notch1 and its ligands are involved in establishing the anterior–posterior orientation from the short bud to long bud stages [56]. Notch1/2 is expressed before epidermal placode formation, and the expression of its ligand, Delta1, can be observed during early bud initiation. The overexpression of Delta1 blocks the expression of Shh, Bmp2, Bmp4, and Bmp7, as well as feather bud initiation in virally infected skin [57]. The overexpression of Delta1 in chicken embryos results in the blocking of feather bud formation, which can be saved through FGF application [58]. This suggests that Notch/Delta can interact with the FGF pathway to promote feather bud formation during early feather follicle morphogenesis. Chen et al. reported that Notch and FGF signaling drives the differential cell adhesion and contraction of basal filopodia to regulate feather branching in regenerated feathers [59]. Whether Notch/FGF signaling can regulate the growth of feather branch formation in embryonic age requires further verification.

#### 2.2.8. Non-coding RNA (ncRNA) Regulation in Feather Follicle Morphogenesis 

Non-coding RNAs are a variety of RNAs that do not translate into proteins and were originally considered by-products of transcription with fewer biological functions. However, recent studies have suggested some of them, such as micro-RNAs (miRNAs), long-noncoding RNAs (lncRNAs), and circular RNAs (circRNAs), act as key regulators in diverse aspects of biological functions, such as chromosome silencing, genomic imprinting, chromatin modification, transcriptional activation, and interference. NcRNAs exist widely in animal tissues and organs and regulate target gene expression via epigenetic regulation, transcriptional regulation, and post-transcriptional levels [60,61]. A growing number of ncRNAs in chickens have been identified and studied in terms of their specific mechanisms via high-throughput sequencing techniques. Among these types of ncRNA, microRNAs and lncRNAs play predominant regulatory roles in poultry feather development.

Bao et al. [62] detected differentially expressed mRNA and miRNA between the scutate scale and dermal and wing feather tissues during chicken embryonic development via microarray technology. The results showed that gga-miR-6651-5p and gga-miR-10b-3p initialized scale development through the repression of FGF20 and the improvement of ALDH1A3 expression in the metatarsal epidermis of chickens at E8. The miRNA gga-miR-146a-5p may play a role in avian epidermal differentiation, such as in the formation of feather barbs and barbules, via the regulation of Tenascin C (TNC) splice variant expression on the avian scutate scale and feather development. Multiple miRNAs, including gga-miR-1556 and gga-miR-204, may contribute to feather barbule morphological diversity by regulating β-keratins that are differentially expressed in feather development. A study on goose embryos found that miR-144-y was able to inhibit the expression of FOXO3, which is a hub gene highly related to feather follicle formation through the regulation of Shh signaling pathways [63]. Chen et al. investigated the miRNA expression profiles in duck feather follicle tissue development at the embryonic and post-hatching stages [64]. The results showed that miRNA expression can be categorized in two expression patterns, namely, the embryonic and early post-hatch stage (4 weeks), and the 18 most highly expressed miRNAs were identified as having their target genes significantly enriched in focal adhesion, i.e., the MAPK, ErbB, Wnt, Notch, and TGF-β signaling pathways. A study on duck feather follicles at 8 weeks post-hatching identified seven important miRNAs that were related to feather follicle formation because their targeted genes were involved in Wnt/β-catenin, Notch, Shh, and BMP pathways [65]. Fang et al. analyzed miRNAs and mRNA expression profiles in chicken wing skin and identified 14 negatively interacting miRNA–mRNA duplexes, which played important roles in feather follicle development and feathering phenotypes [66]. Using RNA sequencing, miR-10b-1-5p, miR-30e-1-5p, and miR-204-5p were found to be predominantly expressed in black feather bulbs and are probably associated with plumage pigmentation [67]. The known information on the roles of microRNAs in feather follicle development is summarized in Table 1.

A study on chicken feather regeneration found that lnc1589, lnc3500, and lnc7831 are able to regulate feather regeneration and axis formation through the inhibition of the Wnt signaling pathway [68]. RNA sequencing results in early/late feathering chickens found hundreds of differently expressed lncRNAs, and their target mRNAs were mainly enriched in the Jak-STAT, Wnt, TGF-β, and calcium signaling pathways [39]. A further dual luciferase reporter assay demonstrated that the ENSGALG00000047626–gga-miR-1649-5p–SSTR2 ceRNA regulatory network plays a crucial role in the feather rate phenotype. In addition, it has been found that lncRNA TCONS_00054154 and lnc-THMEM184c, among others, play key roles in regulating melanogenesis through target genes, such as EDN3, EDNRA, SLMO2, etc., in chicken skin [69,70]. The known information on LncRNAs in feather follicle traits is summarized in Table 2.

It has been found that ncRNAs play significant modulatory roles in hair follicle development through the regulation of Wnt/β-catenin, Shh, BMP, and Notch signaling pathways [72]. As shown above, with the development of sequencing technology and the reduction in costs, more and more miRNAs/LncRNAs related to feather follicle formation have been identified in poultry. However, the biological functions of miRNAs/LncRNAs and the regulation mechanism between miRNAs/LncRNAs and their target genes remain unclear because most of the results still remain in the bioinformatics analysis stage. This may be due to multiple reasons. MiRNAs displayed high evolutionary conservation between species. On the other hand, the conserved sequences of lncRNAs between species are not numerous, resulting in little significance in terms of the mutual borrowing of lncRNA action mechanisms between species. Gain-of-function (overexpression) or loss-of-function (RNA interference, antisense oligonucleotides, or CRISPR system) approaches were often used to establish the function of target genes or ncRNAs, but few stable cell lines have been established in studies related to chicken feather follicles. The expressions of miRNAs/LncRNAs exhibit obvious space and time patterns. At the same time, miRNAs/LncRNAs can execute their functions through the targeting of one or more signaling pathways. The key genes associated with the feather follicle development described above are probably regulated by several types of ncRNAs. This further increases the difficulty of conducting in-depth functional studies on the identified micRNAs/lncRNAs. 

Most miRNAs/LncRNAs target genes identified belong to feather follicle- or feather formation-related signaling pathways, suggesting that complex regulatory networks involving genes and ncRNAs exist in feather follicle development. We should pay more addition to the process of experimentally validating the specific regulatory mechanisms of the discovered miRNAs/LncRNAs in chicken feather follicle formation.

## 3. Association of SNP Sites with Feather Follicle and Feather Traits

Single nucleotide polymorphisms (SNPs) are the simplest forms of genetic markers and are caused by a single nucleotide mutation. They account for more than 90% of all known polymorphisms [73]. SNPs are considered to be the best molecular markers as they are characterized by high density, wide distribution, genetic stability, and suitability for automated high-throughput analysis [74,75]. The SNP genotyping combined with genetic background can improve breeding prediction accuracy and has been widely explored in genetic analysis, such as genome-wide association analysis and animal breeding. Utilizing high-throughput sequencing technology to select genetic markers associated with highly valued qualities, such as meat quality, fat deposition, feed efficiency, and reproductive traits, is becoming more and more common. Rong et al. identified that 13 SNPs of the MTR gene were significantly associated with wool production and wool quality-related traits [76]. Comparative genome results in sheep showed that an SNP (c.-253G>A) in the 5′-UTR of the FGF5 gene induced premature FGF5 protein synthesis and is probably related to the long hair phenotype [77]. The application of whole-genome resequencing and the SNP array revealed that one SNP of the EDAR gene upregulated EDAR mRNA expression by creating a new SOX2 binding site, which is associated with the increased density of hair placodes [78].

In poultry, Feng et al. reported that a C to G transversion significantly reduced PDSS2 expression during feather development, resulting in a silky feather phenotype [79]. It was found that for the Msx2 gene, as a downstream target of BMP signaling, its overexpression significantly increased dermal fibroblast proliferation, and the missense mutations of SNPs located in exon 1 significantly increased the feather follicle diameter of newborn geese [80,81]. Chen et al. compared the diameter and density of feather follicles at different sites from incubation to the birth of goslings and identified two synonymous mutations of SNP sites in the Wnt6 gene [82]; however, it is unclear if these two SNP sites are associated with feather follicle density in geese. The feather growth and maturation phenotypic data were collected in ducks, and it was found that 116 SNPs were related to feather length traits via genome-wide association analysis (GWAS) [83]. Ji et al. reported that an SNP site located in the introns of the Wnt3a gene was associated with the feather follicle density of thigh skin in chickens [24]. A total of 146 SNP markers were identified, which showed significant association with the dorsal feather follicle density in chickens through the GWAS method [84]. These SNPs are closely linked to genes DNAJC15, SUCLA2, MLNR, and DHRS12. Six of the significant SNPs fall into the non-coding regions of the genome, indicating that ncRNAs probably play vital roles in the process of feather follicle development. Regarding chickens, previous studies paid more attention to the association between the plumage color and the genetic variation of key genes because people from different regions have different preferences in regard to the color of chicken feathers [85,86]. It has been found that the mutations of many genes, including MC1R, MCAM, TYR, and ASIP, are significantly associated with the plumage color [87,88,89].

Overall, there are only a few studies related to feather follicle density and diameter size in chickens in comparison with those on hair follicles. A large number of SNP sites that are associated with feather follicles or feather traits were found, but the function of the candidate genes is still unclear. There are few relevant works that report on the differences in feather follicle density between chicken breeds. The measurement methods of feather follicle density or diameter can be divided into two categories: histopathologic section or direct count within a 2 × 2 cm^2^ area in different sections. More research is needed to determine the suitability of detecting the feather follicle density in chickens. Compared with the known SNP molecular markers identified in hair follicles, how the SNPs control, interact with, or regulate gene expression and the different degrees of correlation with the core genes identified in the feather follicle development process remain unclear. Further studies need to be carried out to confirm the relationships between SNP markers and feather follicle traits in chickens.

## 4. Conclusions

Feather follicle morphogenesis is completed during the embryonic stage. The Wnt, EGF, Eda, FGF, Shh, and Notch/Delta pathways are positive pathways, and the BMP pathway has negative roles and is involved in feather follicle development. The crosstalk between these factors in the feather follicle formation process still needs to be investigated further. The ncRNAs collaborate with genes of the Wnt, Shh, BMP, and Notch pathways to form a complex regulatory network, which regulates feather follicle growth and development. Most studies focus on the temporal changes in the feather follicle growth and development processes. There is little research on the differences in feather follicle traits and gene expression among the different chicken breeds. Few SNP sites have been found to be closely related to feather follicle traits. It is necessary to further study the molecular regulatory mechanisms of feather follicle traits to provide a theoretical basis for novel interventions that can enhance the carcass appearance for the commercial sale of yellow-feathered broilers.

## Figures and Tables

**Table 1:** MicroRNAs involved in feather development of poultry.

Name	Expression Location	Targen Genes	Function	Days of Age	Target Binding Location	Reference
gga-miR-10b-3p	Dorsa skin	ALDH1A3	Feather placode development ↓	E8	Coding region	[62]
gga-miR-6679-5p	Scale skin	EDAR	Not mentioned	E17	Coding region	
gga-miR-146a-5p	Scale skin	TNC	epidermal differentiation ↓	E17	Coding region	
gga-miR-6651-5p	Scale skin	FGF20	Feather placode development ↓	E8	Coding region	
gga-miR-204	Dorsa skin	β-keratins	Epidermal differentiation -	E17 E19	Coding region	
gga-miR-1806	Wing skin	β-keratins	Epidermal differentiation -		Coding region	
gga-miR-1556	Dorsa skin	β-keratins	Epidermal differentiation -		Coding region	
gga-miR-1759-3p	Scale skin	GBX2	Not mentioned		Coding region	
gga-miR-100-5p, etc.	Breast skin	HS3ST2, PIGT, etc.	Feather follicle formation -	E11, E15, E20, D1, D4, 10 W	Not mentioned	[64]
miR-204 -5p	Feather bulbs	PRKCA, LEF1, KIT	Plumage pigmentation -	Not mentioned	Not mentioned	[67]
miR-27b -3p	Feather bulbs	MITF				
miR-128	Feather bulbs	PLCB2				
miR-222	Feather bulbs	RAF1, CTNNB1				
miR-130a	Feather bulbs	ADCY2				
miR-30e	Black feather bulbs	MITF				
miR-22-3p	Feather bulbs	GNAO1, CALM, CAMK2D				
miR-15-1-5p	Feather bulbs	PRKCA				
miR-99a-5p	Feather bulbs	PLCB2				
gga-miR-130b-5p	Wing skin	ERG	Cell differentiation -	Not mentioned	Not mentioned	[66]
gga-miR-34a-5p	Wing skin	ERBB4	Cell fate commitment -			
gga-miR-216a	Wing skin	KRT6A	Epithelial cell differentiation-			
gga-miR-1574-5p	Wing skin	NR2F2	Limb development -			
gga-miR-193a-5p	Wing skin	PRRX1	Embryonic limb morphogenesis -			
gga-miR-365-2-5p	Wing skin	CDK6, JAK3	Osteoblast differentiation -			
miR-144-y	Feather bud	FOXO3	Skin and feather follicles growth -	E10–E28	3’UTR	[63]
miR-107, etc.	Feather follicle	Wnt, Shh, BMP Notch pathways	Feather follicle formation -	8 W	Not mentioned	[65]

↓: inhibit, -: unknown; E: embryonic age; W: week.

**Table 2:** LncRNAs involved in feather development of poultry.

lncRNA	Loctation	Target Gene	Function	Target Binding Location	Reference
lnc5351	Epithelium	Wnt ligands	Unclear	Not mentioned	[68]
lnc7349	Epithelium	Wnt ligands	Unclear		
lnc1589	Dermal papillae	Wnt inhibitor	Feather regeneration and axis formation ↓		
lnc3500	Dermal papillae	Wnt inhibitor	Feather regeneration and axis formation ↓		
lnc7831	Dermal papillae	Wnt inhibitor	Feather regeneration and axis formation ↓		
ENSGALG00000047626	Feather follicle	SSTR2	Feather rate phenotype -	gga-miR-1649-5p	[71]
TCONS_00054154	Black skin	EDN3,SLMO2, ATP5E	Melanogenesis ↑	Not mentioned	[69]
linc-THEM184c	Black skin	EDNRA., etc	Melanogenesis ↑	Promoter	[70]

↑: promote, ↓: inhibit, -: unknown.

## Data Availability

Not applicable.

## References

[1] USDA (2023). Livestock and Poultry:World Markets and Trade.

[2] Devatkal S.K., Naveena B.M., Kotaiah T. (2019). Quality, composition, and consumer evaluation of meat from slow-growing broilers relative to commercial broilers. Poult. Sci..

[3] Jiang M., Fan W.L., Xing S.Y., Wang J., Li P., Liu R.R., Li Q.H., Zheng M.Q., Cui H.X., Wen J. (2017). Effects of balanced selection for intramuscular fat and abdominal fat percentage and estimates of genetic parameters. Poult. Sci..

[4] Yuan C., Jiang Y., Wang Z., Chen G., Bai H., Chang G. (2022). Indigenous, Yellow-Feathered Chickens Body Measurements, Carcass Traits, and Meat Quality Depending on Marketable Age. Animals.

[5] Xu H.M., Zhang K.Y., Bai S.P., Ding X.M., Wang J.P., Peng H.W., Xuan Y., Su Z.W., Gang T., Zeng Q.F. (2021). Dietary resistant potato starch improves growth performance and feather development in Pekin ducks fed a low phosphorus diet. Poult. Sci..

[6] Yin L.Y., Wang Z.Y., Yang H.M., Xu L., Zhang J., Xing H. (2017). Effects of stocking density on growth performance, feather growth, intestinal development, and serum parameters of geese. Poult. Sci..

[7] Yu M., Yue Z., Wu P., Wu D.Y., Mayer J.A., Medina M., Widelitz R.B., Jiang T.X., Chuong C.M. (2004). The biology of feather follicles. Int. J. Dev. Biol..

[8] Lin X., Zhu L., He J. (2022). Morphogenesis, Growth Cycle and Molecular Regulation of Hair Follicles. Front. Cell. Dev. Biol..

[9] Yu M., Wu P., Widelitz R.B., Chuong C.M. (2002). The morphogenesis of feathers. Nature.

[10] Xie W.Y., Chen M.J., Jiang S.G., Yan H.C., Wang X.Q., Gao C.Q. (2020). Investigation of feather follicle morphogenesis and the expression of the Wnt/beta-catenin signaling pathway in yellow-feathered broiler chick embryos. Br. Poult. Sci..

[11] Wu P., Yan J., Lai Y.C., Ng C.S., Li A., Jiang X., Elsey R.M., Widelitz R., Bajpai R., Li W.H. (2018). Multiple Regulatory Modules Are Required for Scale-to-Feather Conversion. Mol. Biol. Evol..

[12] Lin C.M., Yuan Y.P., Chen X.C., Li H.H., Cai B.Z., Liu Y., Zhang H., Li Y., Huang K. (2015). Expression of Wnt/beta-catenin signaling, stem-cell markers and proliferating cell markers in rat whisker hair follicles. J. Mol. Histol..

[13] Chang C.H., Jiang T.X., Lin C.M., Burrus L.W., Chuong C.M., Widelitz R. (2004). Distinct Wnt members regulate the hierarchical morphogenesis of skin regions (spinal tract) and individual feathers. Mech. Dev..

[14] Lai Y., Liang Y., Jiang T., Widelitz R.B., Chuong C. (2018). Transcriptome analyses of reprogrammed feather/scale chimeric explants revealed co-expressed epithelial gene networks during organ specification. BMC Genom..

[15] Widelitz R.B., Jiang T.X., Lu J., Chuong C.M. (2000). beta-catenin in epithelial morphogenesis: Conversion of part of avian foot scales into feather buds with a mutated beta-catenin. Dev. Biol..

[16] Feng Z., Mabrouk I., Msuthwana P., Zhou Y., Song Y., Gong H., Li S., Min C., Ju A., Duan A. (2022). In ovo injection of CHIR-99021 promotes feather follicles development via activating Wnt/beta-catenin signaling pathway during chick embryonic period. Poult. Sci..

[17] Feng Z., Gong H., Fu J., Xu X., Song Y., Yan X., Mabrouk I., Zhou Y., Wang Y., Fu X. (2022). In Ovo Injection of CHIR-99021 Promotes Feather Follicle Development via Modulating the Wnt Signaling Pathway and Transcriptome in Goose Embryos (Anser cygnoides). Front. Physiol..

[18] Tripurani S.K., Wang Y., Fan Y.X., Rahimi M., Wong L., Lee M.H., Starost M.F., Rubin J.S., Johnson G.R. (2018). Suppression of Wnt/beta-catenin signaling by EGF receptor is required for hair follicle development. Mol. Biol. Cell..

[19] Zhang H., Nan W., Wang S., Si H., Li G. (2018). Balance between fibroblast growth factor 10 and secreted frizzled-relate protein-1 controls the development of hair follicle by competitively regulating beta-catenin signaling. Biomed. Pharmacother..

[20] Hu X., Zhang X., Liu Z., Li S., Zheng X., Nie Y., Tao Y., Zhou X., Wu W., Yang G. (2020). Exploration of key regulators driving primary feather follicle induction in goose skin. Gene.

[21] Xie W.Y., Chen M.J., Jiang S.G., Yan H.C., Wang X.Q., Gao C.Q. (2020). The Wnt/β-catenin signaling pathway is involved in regulating feather growth of embryonic chicks. Poult. Sci..

[22] Sick S., Reinker S., Timmer J., Schlake T. (2006). WNT and DKK determine hair follicle spacing through a reaction-diffusion mechanism. Science.

[23] Ji G.G., Zhang M., Liu Y.F., Shan Y.J., Tu Y.J., Ju X.J., Zou J.M., Shu J.T., Wu J.F., Xie J.F. (2021). A gene co-expression network analysis of the candidate genes and molecular pathways associated with feather follicle traits of chicken skin. J. Anim. Breed Genet..

[24] Chae W.J., Bothwell A. (2018). Canonical and Non-Canonical Wnt Signaling in Immune Cells. Trends Immunol..

[25] Chodankar R., Chang C.H., Yue Z., Jiang T.X., Suksaweang S., Burrus L., Chuong C.M., Widelitz R. (2003). Shift of localized growth zones contributes to skin appendage morphogenesis: Role of the Wnt/beta-catenin pathway. J. Invest. Dermatol..

[26] Chuong C.M., Widelitz R.B., Ting-Berreth S., Jiang T.X. (1996). Early events during avian skin appendage regeneration: Dependence on epithelial-mesenchymal interaction and order of molecular reappearance. J. Investig. Dermatol..

[27] Gong H., Wang H., Wang Y., Bai X., Liu B., He J., Wu J., Qi W., Zhang W. (2018). Skin transcriptome reveals the dynamic changes in the Wnt pathway during integument morphogenesis of chick embryos. PLoS ONE.

[28] Arbouzova N., Mcneill H. (2008). Visualization of PCP defects in the eye and wing of Drosophila melanogaster. Methods Mol. Biol..

[29] Al M.A., Achkhar A., Yasmeen A. (2012). EGF-receptor signaling and epithelial-mesenchymal transition in human carcinomas. Front. Biosci. (Schol Ed)..

[30] Atit R., Conlon R.A., Niswander L. (2003). EGF signaling patterns the feather array by promoting the interbud fate. Dev. Cell.

[31] Ornitz D.M., Itoh N. (2015). The Fibroblast Growth Factor signaling pathway. Wires Dev. Biol..

[32] Widelitz R.B., Jiang T., Noveen A., Chen C.J., Chuong C. (1996). FGF induces new feather buds from developing avian skin. J. Invest. Dermatol..

[33] Khan S.A., Rukan A., Ullah A., Bibi N., Humayun M., Ullah W., Raza R., Muhammad N., Ahmad W., Khan S. (2020). Homozygous variants of EDAR underlying hypohidrotic ectodermal dysplasia in three consanguineous families. Eur. J. Dermatol..

[34] Yang R., Mei Y., Jiang Y., Li H., Zhao R., Sima J., Yao Y. (2022). Ectodysplasin A (EDA) Signaling: From Skin Appendage to Multiple Diseases. Int. J. Mol. Sci..

[35] Houghton L., Lindon C., Morgan B.A. (2005). The ectodysplasin pathway in feather tract development. Development.

[36] Ho W.K., Freem L., Zhao D., Painter K.J., Woolley T.E., Gaffney E.A., Mcgrew M.J., Tzika A., Milinkovitch M.C., Schneider P. (2019). Feather arrays are patterned by interacting signalling and cell density waves. PLoS Biol..

[37] Drew C.F., Lin C.M., Jiang T.X., Blunt G., Mou C., Chuong C.M., Headon D.J. (2007). The Edar subfamily in feather placode formation. Dev. Biol..

[38] Wu Z., Wang Y., Han W., Yang K., Hai E., Ma R., Di Z., Shang F., Su R., Wang R. (2020). EDA and EDAR expression at different stages of hair follicle development in cashmere goats and effects on expression of related genes. Arch. Tierz..

[39] Kondo S., Kuwahara Y., Kondo M., Naruse K., Mitani H., Wakamatsu Y., Ozato K., Asakawa S., Shimizu N., Shima A. (2001). The medaka rs-3 locus required for scale development encodes ectodysplasin-A receptor. Curr. Biol..

[40] Botchkarev V.A. (2003). Bone morphogenetic proteins and their antagonists in skin and hair follicle biology. J. Invest. Dermatol..

[41] Liu M., Goldman G., Macdougall M., Chen S. (2022). BMP Signaling Pathway in Dentin Development and Diseases. Cells.

[42] Ashique A.M., Fu K., Richman J.M. (2002). Signalling via type IA and type IB bone morphogenetic protein receptors (BMPR) regulates intramembranous bone formation, chondrogenesis and feather formation in the chicken embryo. Int. J. Dev. Biol..

[43] Jung H.S., Francis-West P.H., Widelitz R.B., Jiang T.X., Ting-Berreth S., Tickle C., Wolpert L., Chuong C.M. (1998). Local inhibitory action of BMPs and their relationships with activators in feather formation: Implications for periodic patterning. Dev. Biol..

[44] Scaal M., Pröls F., Füchtbauer E.M., Patel K., Hornik C., Köhler T., Christ B., Brand-Saberi B. (2002). BMPs induce dermal markers and ectopic feather tracts. Mech. Dev..

[45] Michon F., Forest L., Collomb E., Demongeot J., Dhouailly D. (2008). BMP2 and BMP7 play antagonistic roles in feather induction. Development.

[46] Patel K., Makarenkova H., Jung H.S. (1999). The role of long range, local and direct signalling molecules during chick feather bud development involving the BMPs, follistatin and the Eph receptor tyrosine kinase Eph-A4. Mech. Dev..

[47] Liu A. (2019). Proteostasis in the Hedgehog signaling pathway. Semin. Cell. Dev. Biol..

[48] Mckinnell I.W., Turmaine M., Patel K. (2004). Sonic Hedgehog functions by localizing the region of proliferation in early developing feather buds. Dev. Biol..

[49] Li A., Cho J.H., Reid B., Tseng C.C., He L., Tan P., Yeh C.Y., Wu P., Li Y., Widelitz R.B. (2018). Calcium oscillations coordinate feather mesenchymal cell movement by SHH dependent modulation of gap junction networks. Nat. Commun..

[50] Harris M.P., Fallon J.F., Prum R.O. (2002). Shh-Bmp2 signaling module and the evolutionary origin and diversification of feathers. J. Exp. Zool..

[51] Harris M.P., Williamson S., Fallon J.F., Meinhardt H., Prum R.O. (2005). Molecular evidence for an activator-inhibitor mechanism in development of embryonic feather branching. Proc. Natl. Acad. Sci. USA.

[52] Artavanis-Tsakonas S., Rand M.D., Lake R.J. (1999). Notch signaling: Cell fate control and signal integration in development. Science.

[53] Andersson E.R., Sandberg R., Lendahl U. (2011). Notch signaling: Simplicity in design, versatility in function. Development.

[54] Del G.F., Liu D., Lendahl U. (2022). Notch signalling in healthy and diseased vasculature. Open Biol..

[55] Kopan R., Ilagan M.X. (2009). The canonical Notch signaling pathway: Unfolding the activation mechanism. Cell.

[56] Chen C.W., Jung H.S., Jiang T.X., Chuong C.M. (1997). Asymmetric expression of Notch/Delta/Serrate is associated with the anterior-posterior axis of feather buds. Dev. Biol..

[57] Crowe R., Henrique D., Ish-Horowicz D., Niswander L. (1998). A new role for Notch and Delta in cell fate decisions: Patterning the feather array. Development.

[58] Viallet J.P., Prin F., Olivera-Martinez I., Hirsinger E., Pourquie O., Dhouailly D. (1998). Chick Delta-1 gene expression and the formation of the feather primordia. Mech. Dev..

[59] Cheng D., Yan X., Qiu G., Zhang J., Wang H., Feng T., Tian Y., Xu H., Wang M., He W. (2018). Contraction of basal filopodia controls periodic feather branching via Notch and FGF signaling. Nat. Commun..

[60] Quan M., Chen J., Zhang D. (2015). Exploring the secrets of long noncoding RNAs. Int. J. Mol. Sci..

[61] Panni S., Lovering R.C., Porras P., Orchard S. (2020). Non-coding RNA regulatory networks. Bba-Gene Regul. Mech..

[62] Bao W., Greenwold M.J., Sawyer R.H. (2016). Expressed miRNAs target feather related mRNAs involved in cell signaling, cell adhesion and structure during chicken epidermal development. Gene.

[63] Mabrouk I., Zhou Y., Wang S., Song Y., Fu X., Xu X., Liu T., Wang Y., Feng Z., Fu J. (2022). Transcriptional Characteristics Showed That miR-144-y/FOXO3 Participates in Embryonic Skin and Feather Follicle Development in Zhedong White Goose. Animals.

[64] Chen X., Ge K., Wang M., Zhang C., Geng Z. (2017). Integrative analysis of the Pekin duck (Anas anas) MicroRNAome during feather follicle development. BMC Dev. Biol..

[65] Zhang L., Nie Q., Su Y., Xie X., Luo W., Jia X., Zhang X. (2013). MicroRNA profile analysis on duck feather follicle and skin with high-throughput sequencing technology. Gene.

[66] Fang G., Jia X., Li H., Tan S., Nie Q., Yu H., Yang Y. (2018). Characterization of microRNA and mRNA expression profiles in skin tissue between early-feathering and late-feathering chickens. BMC Genom..

[67] Apopo S., Liu H., Jing L., Du X., Xie S., Gong Y., Xu R., Li S. (2015). Identification and profiling of microRNAs associated with white and black plumage pigmentation in the white and black feather bulbs of ducks by RNA sequencing. Anim. Genet..

[68] Lin X., Gao Q.X., Zhu L.Y., Zhou G.X., Ni S.W., Han H., Yue Z.C. (2018). Long noncoding RNAs regulate Wnt signaling during feather regeneration. Development.

[69] Zhang P., Cao Y., Fu Y., Zhu H., Xu S., Zhang Y., Li W., Sun G., Jiang R., Han R. (2022). Revealing the Regulatory Mechanism of lncRNA-LMEP on Melanin Deposition Based on High-Throughput Sequencing in Xichuan Chicken Skin. Genes.

[70] Hong H., Chai H.-H., Nam K., Lim D., Lee K.-T., Do Y.J., Cho C.-Y., Nam J.-W. (2018). Non-coding transcriptome maps across twenty tissues of the Korean Black Chicken, Yeonsan Ogye. Int. J. Mol. Sci..

[71] Qiu M., Yu C., Zhu S., Liu S., Peng H., Xiong X., Chen J., Jiang X., Du H., Li Q. (2022). RNA sequencing reveals lncRNA-mediated non-mendelian inheritance of feather growth change in chickens. Genes Genom..

[72] Yang M., Weng T., Zhang W., Zhang M., He X., Han C., Wang X. (2021). The Roles of Non-coding RNA in the Development and Regeneration of Hair Follicles: Current Status and Further Perspectives. Front. Cell. Dev. Biol..

[73] Shastry B.S. (2009). SNPs: Impact on gene function and phenotype. Methods Mol. Biol..

[74] Yang W., Kang X., Yang Q., Lin Y., Fang M. (2013). Review on the development of genotyping methods for assessing farm animal diversity. J. Anim. Sci. Biotechnol..

[75] Vignal A., Milan D., Sancristobal M., Eggen A. (2002). A review on SNP and other types of molecular markers and their use in animal genetics. Genet. Sel. Evol..

[76] Rong E.G., Yang H., Zhang Z.W., Wang Z.P., Yan X.H., Li H., Wang N. (2015). Association of methionine synthase gene polymorphisms with wool production and quality traits in Chinese Merino population. J. Anim. Sci..

[77] Guo J., Zhong J., Li L., Zhong T., Wang L., Song T., Zhang H. (2019). Comparative genome analyses reveal the unique genetic composition and selection signals underlying the phenotypic characteristics of three Chinese domestic goat breeds. Genet. Sel. Evol..

[78] Liang B., Bai T., Zhao Y., Han J., He X., Pu Y., Wang C., Liu W., Ma Q., Tian K. (2023). Two mutations at KRT74 and EDAR synergistically drive the fine-wool production in Chinese sheep. J. Adv. Res..

[79] Feng C., Gao Y., Dorshorst B., Song C., Li N. (2014). A cis-Regulatory Mutation of PDSS2 Causes Silky-Feather in Chickens. PLoS Genet..

[80] Song Y., Liu C., Zhou Y., Lin G., Xu C., Msuthwana P., Wang S., Ma J., Zhuang F., Fu X. (2022). Regulation of feather follicle development and Msx2 gene SNP degradation in Hungarian white goose. BMC Genom..

[81] Wu Q., Zhang L., Su P., Lei X., Liu X., Wang H., Lu L., Bai Y., Xiong T., Li D. (2015). MSX2 mediates entry of human pluripotent stem cells into mesendoderm by simultaneously suppressing SOX2 and activating NODAL signaling. Cell Res..

[82] Chen X., Bai H., Li L., Zhang W., Jiang R., Geng Z. (2012). Follicle characteristics and follicle developmental related Wnt6 polymorphism in Chinese indigenous Wanxi-white goose. Mol. Biol. Rep..

[83] Liu X., Zhou R., Peng Y., Zhang C., Li L., Lu C., Li X. (2019). Feather follicles transcriptome profiles in Bashang long-tailed chickens with different plumage colors. Genes Genomics.

[84] Sun H., Hu Y., Dou T., Qu L., Ma M., Lu J., Wang X., Shen M., Wang K. (2019). Genetic architecture related to contour feathers density in an F(2) resource population via a genome-wide association study. 3 Biotech.

[85] Wang H., Wen J., Li H., Zhu T., Zhao X., Zhang J., Zhang X., Tang C., Qu L., Gemingguli M. (2022). Candidate pigmentation genes related to feather color variation in an indigenous chicken breed revealed by whole genome data. Front. Genet..

[86] Davoodi P., Ehsani A., Vaez T.R., Masoudi A.A. (2022). New insights into genetics underlying of plumage color. Anim. Genet..

[87] Nie C., Qu L., Li X., Jiang Z., Wang K., Li H., Wang H., Qu C., Qu L., Ning Z. (2021). Genomic Regions Related to White/Black Tail Feather Color in Dwarf Chickens Identified Using a Genome-Wide Association Study. Front. Genet..

[88] Yang C.W., Ran J.S., Yu C.L., Qiu M.H., Zhang Z.R., Du H.R., Li Q.Y., Xiong X., Song X.Y., Xia B. (2019). Polymorphism in MC1R, TYR and ASIP genes in different colored feather chickens. 3 Biotech..

[89] Fan Y., Wu X., Li Y., Han H., Zhang Y., Yang J., Liu Y. (2022). Effect of polymorphisms in the 5′-flanking sequence of MC1R on feather color in Taihang chickens. Poult. Sci..

